# Author Correction: A One Health framework for exploring zoonotic interactions demonstrated through a case study

**DOI:** 10.1038/s41467-024-51222-y

**Published:** 2024-08-07

**Authors:** Amélie Desvars-Larrive, Anna Elisabeth Vogl, Gavrila Amadea Puspitarani, Liuhuaying Yang, Anja Joachim, Annemarie Käsbohrer

**Affiliations:** 1 Centre for Food Science and Veterinary Public Health, Clinical Department for Farm Animals and Food System Science, University of Veterinary Medicine Vienna, Vienna, Austria; 2 Complexity Science Hub, Vienna, Austria; 3 Centre of Pathobiology, Department of Biological Sciences and Pathobiology, University of Veterinary Medicine Vienna, Vienna, Austria

**Keywords:** Bacterial infection, Viral infection, Parasitic infection, Ecological networks, Epidemiology

Correction to: *Nature Communications* 10.1038/s41467-024-49967-7, published online 15 July 2024

The original version of this Article contained errors in some values quoted in the introduction. The sentence “The country also counts ~53,300 cattle, one million pigs, and five million poultry, while 133,000 hunting permits are issued annually^[27]^.” should have read “The country also counts ~1.8 million cattle, 2.5 million pigs, and over 100 million poultry are slaughtered annually. Additionally, around 133,000 hunting permits are issued each year^[27]^.”. These changes have been made in the PDF and HTML versions of the Article.

