# Supplementary material for: A One Health framework for exploring zoonotic interactions demonstrated through a case study
Source: Nat Commun. 2024 Jul 15;15:5650. doi: 10.1038/s41467-024-49967-7 (PMC11250852; doi:10.1038/s41467-024-49967-7)

Supplementary Information for:

A One Health framework for exploring zoonotic interactions demonstrated through a case study

Amélie Desvars-Larrive[1,2]*, Anna Elisabeth Vogl[1], Gavrila Amadea Puspitarani[1,2], Liuhuaying Yang[2], Anja Joachim[3], Annemarie Käsbohrer[1]

[1] Centre for Food Science and Veterinary Public Health, Clinical Department for Farm Animals and Food System Science, University of Veterinary Medicine Vienna, Vienna, Austria

[2] Complexity Science Hub, Vienna, Austria

[3] Centre of Pathobiology, Department of Biological Sciences and Pathobiology, University of Veterinary Medicine Vienna, Vienna, Austria

Correspondence to Amélie Desvars-Larrive (amelie.desvars@vetmeduni.ac.at)

This file includes Supplementary Figures 1 to 13 and Supplementary Tables 1 to 8.

Supplementary Figure 1: PRISMA flow diagram of publication retrieval and selection. After screening 2,186 records, we synthesised 246 studies reporting direct or indirect evidence of natural zoonotic infections (or contaminations) in humans, animals, food, and environmental matrices in Austria, 1975-2022.

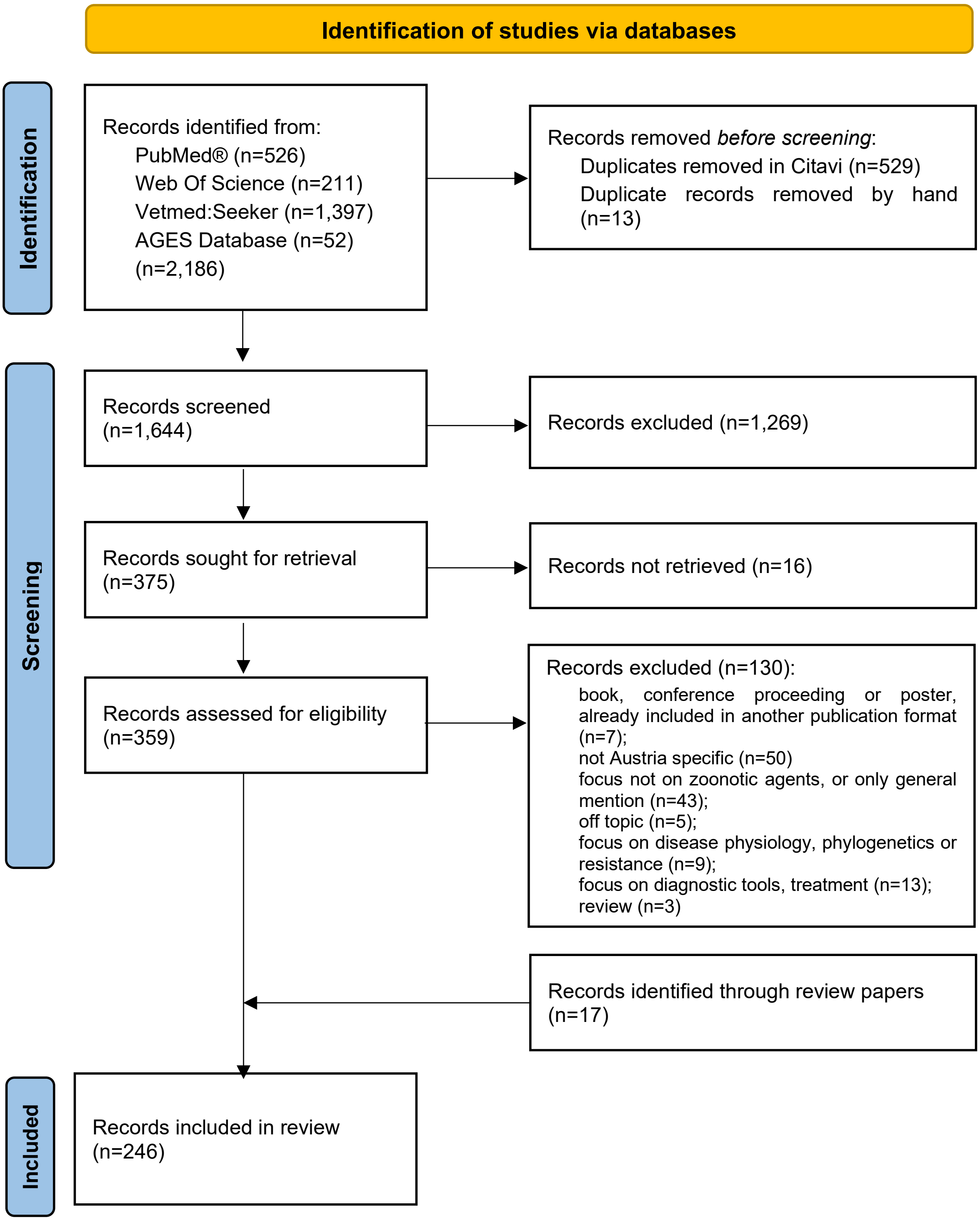

Flow diagram adapted from the template available at: http://www.prisma-statement.org/PRISMAStatement/FlowDiagram.

Supplementary Figure 2: Stacked chart of the annual number of publications that investigated naturally occurring zoonotic agents in Austria between January 1975 and August 2022. Colours represent the superkingdom to which the zoonotic agents belong.

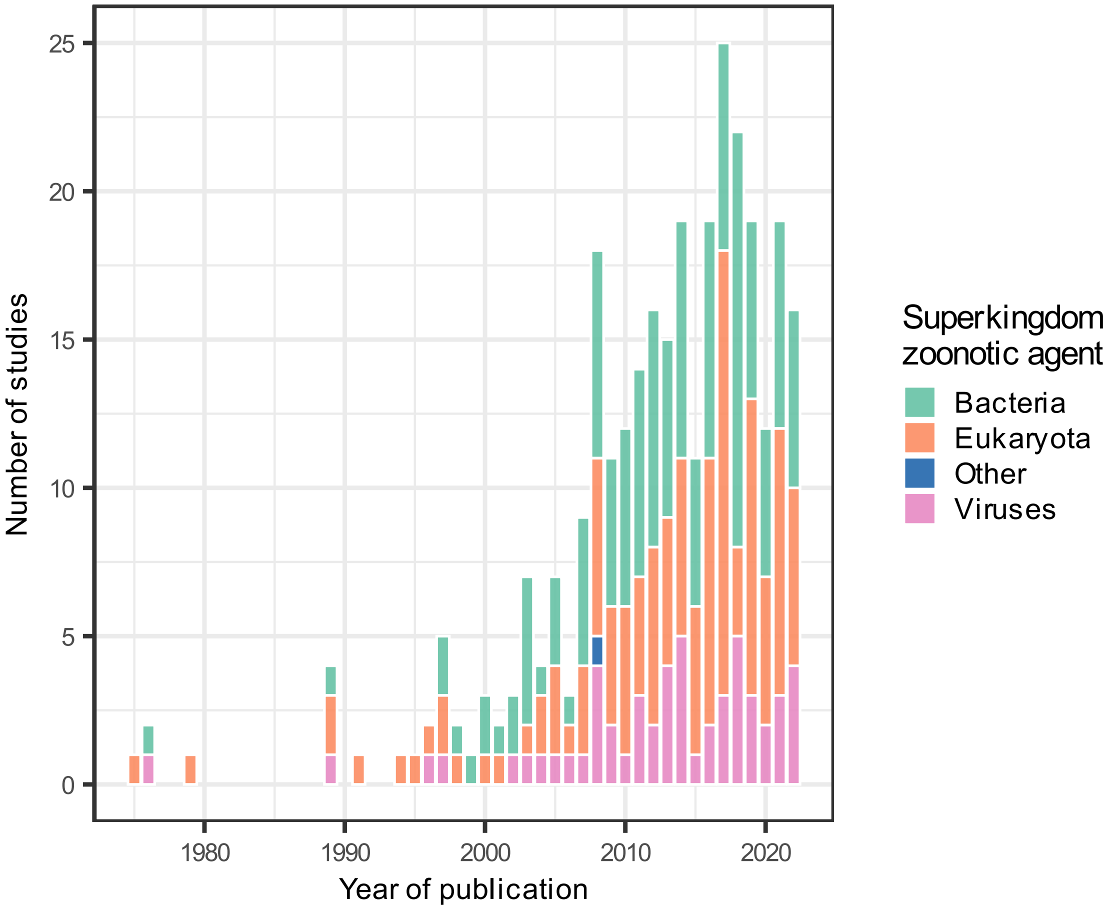

Supplementary Figure 3: Number of publications per zoonotic agent superkingdom and Austrian federal state, Austria, 1975-2022.

Studies conducted at a national scale are represented under “Austria”. The percentage of publications per zoonotic agent superkingdom within each federal state is indicated. Publications that did not specify the federal state (e.g., mentioning “eastern Austria”, “west Austria”, “southwestern Austria”, “southeastern Austria”, or “northern limestone alps”) are not represented. One publication may investigate one or more than one zoonotic agent in one or more than one location.

Twenty-three publications (9.4%) explored zoonotic diseases in multiple countries including Austria, often focusing on cross-border research within a European context. Nationwide studies accounted for 11.4% (n=45) of the selected publications. The highest number of publications (n=68, 17.3%) was carried out in Lower Austria, and the lowest number in Carinthia (n=12, 3%). Information about the exact location of the study was missing in 47 publications.

The federal states in the east of Austria (Burgenland, Lower Austria, and Vienna) as well as Styria showed a higher number of publications, compared to western states. This observation may be attributed to the geographical proximity of the Eastern states to Vienna and Mödling (Lower Austria), where the University of Veterinary Medicine and the Austrian Agency for Health and Food Safety (AGES), respectively, have established their laboratory facilities.

The data revealed fluctuating levels of interest in the types of investigated zoonotic agent, depending on the federal state. The highest difference was observed with viral agents; specifically, Vorarlberg exhibited the lowest percentage of publications discussing viral zoonotic agents with 3% of the studies conducted in this federal state addressing viruses. In contrast, 33% of studies conducted in Lower Austria discussed viral zoonotic agents.

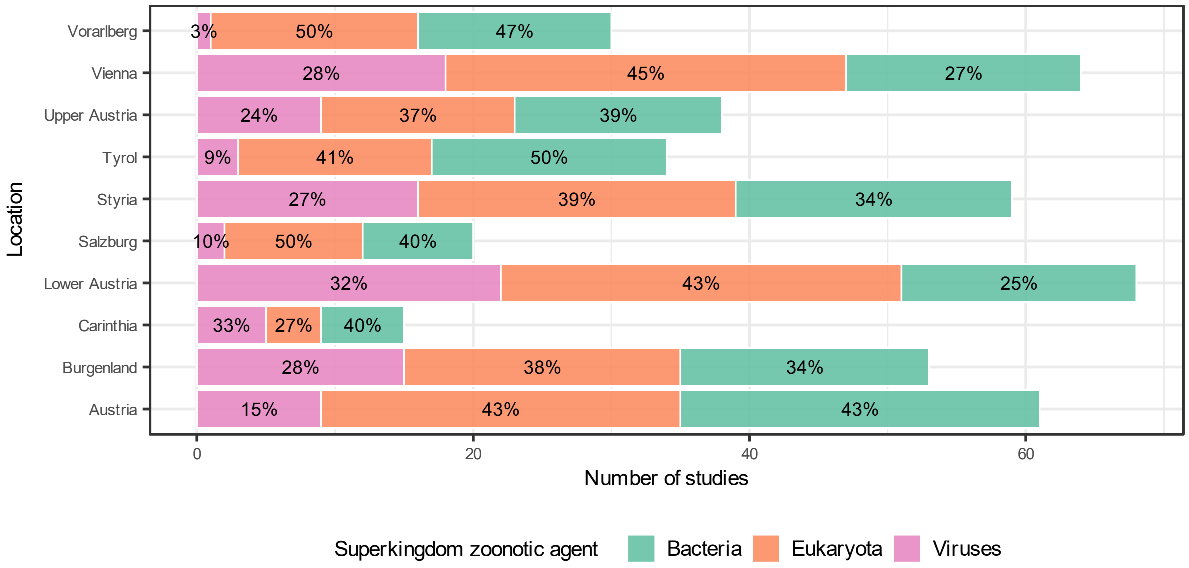

Supplementary Table 1: Zoonotic agent genera investigated in Austria, 1975-2022, ranked by the number of studies.

Genus zoonotic agent	Number of studies	Percentage of the total number of studies
Salmonella	37	5.8
Escherichia	30	4.7
Listeria	28	4.4
Echinococcus	27	4.3
Orthoflavivirus	26	4.1
Brucella	25	3.9
Toxoplasma	24	3.8
Campylobacter	22	3.5
Trichinella	22	3.5
Leptospira	21	3.3
Toxocara	19	3
Rickettsia	18	2.8
Mycobacterium	16	2.5
Babesia	15	2.4
Coxiella	14	2.2
Giardia	14	2.2
Chlamydia	13	2.1
Cryptosporidium	12	1.9
Dirofilaria	12	1.9
Staphylococcus	12	1.9
Anaplasma	11	1.7
Bartonella	11	1.7
Francisella	11	1.7
Trichuris	10	1.6
Alphainfluenzavirus	8	1.3
Borreliella	8	1.3
Ascaris	7	1.1
Orthohantavirus	7	1.1
Fasciola	6	0.9
Leishmania	6	0.9
Paslahepevirus	6	0.9
Encephalitozoon	5	0.8
Ancylostoma	4	0.6
Candidatus Neoehrlichia	4	0.6
Capillaria	4	0.6
Clostridioides	4	0.6

Dipylidium	4	0.6
Ehrlichia	4	0.6
Filarioidea	4	0.6
Orthopoxvirus	4	0.6
Trypanosomatida	4	0.6
Uncinaria	4	0.6
Yersinia	4	0.6
Alaria	3	0.5
Anaplasmataceae	3	0.5
Cardiovirus	3	0.5
Clostridium	3	0.5
Enterococcus	3	0.5
Orthobunyavirus	3	0.5
Streptococcus	3	0.5
Taenia	3	0.5
Toxascaris	3	0.5
Anisakis	2	0.3
Aphthovirus	2	0.3
Baylisascaris	2	0.3
Borna disease virus	2	0.3
Borrelia	2	0.3
Flavivirus	2	0.3
Lyssavirus	2	0.3
Mammarenavirus	2	0.3
Mycoplasma	2	0.3
Onchocerca	2	0.3
Parapoxvirus	2	0.3
Piroplasmida	2	0.3
Shigella	2	0.3
Theileria	2	0.3
Alphamesonivirus	1	0.2
Alphavirus	1	0.2
Ancylostomatidae	1	0.2
Cheyletiella	1	0.2
Chlamydiaceae	1	0.2
Chordopoxvirinae	1	0.2
Cordylobia	1	0.2
Coronavirus	1	0.2
Corynebacterium	1	0.2

Cryptococcus	1	0.2
Dicrocoelium	1	0.2
Erbovirus	1	0.2
Herpesvirus	1	0.2
Hymenolepis	1	0.2
Isospora	1	0.2
Isthmiophora	1	0.2
Mastadenovirus	1	0.2
Mesocestoides	1	0.2
Molineus	1	0.2
Mycolicibacterium	1	0.2
Orthonairovirus	1	0.2
Orthopneumovirus	1	0.2
Parainfluenza virus 4	1	0.2
Pentastomida	1	0.2
Proteus	1	0.2
Rotavirus	1	0.2
Sarcocystis	1	0.2
Schistosoma	1	0.2
Strongyloides	1	0.2
Thelazia	1	0.2
Tritrichomonas	1	0.2
Trueperella	1	0.2

Supplementary Figure 4: Trend in research interest on zoonotic agents in Austria, 1975-2022, within the environmental compartment, as measured by the number of investigations. Dots represent the number of investigations per year; solid lines show fitted trend (loess regression); shaded areas represent the corresponding 95% confidence interval.

Figure 1b (Main Manuscript) shows trends in research interest on zoonotic agents in Austria, 1975-2022, for each One Health compartment, as defined in the traditional One Health view. In this analysis, plant-based foodstuffs, invertebrate vectors, and environmental matrices were grouped under the compartment "environment". Supplementary Figure 4 disaggregates this data, offering a detailed breakdown of the categories within the environmental compartment, enabling a more specific analysis of the observed trend in the number of investigations.

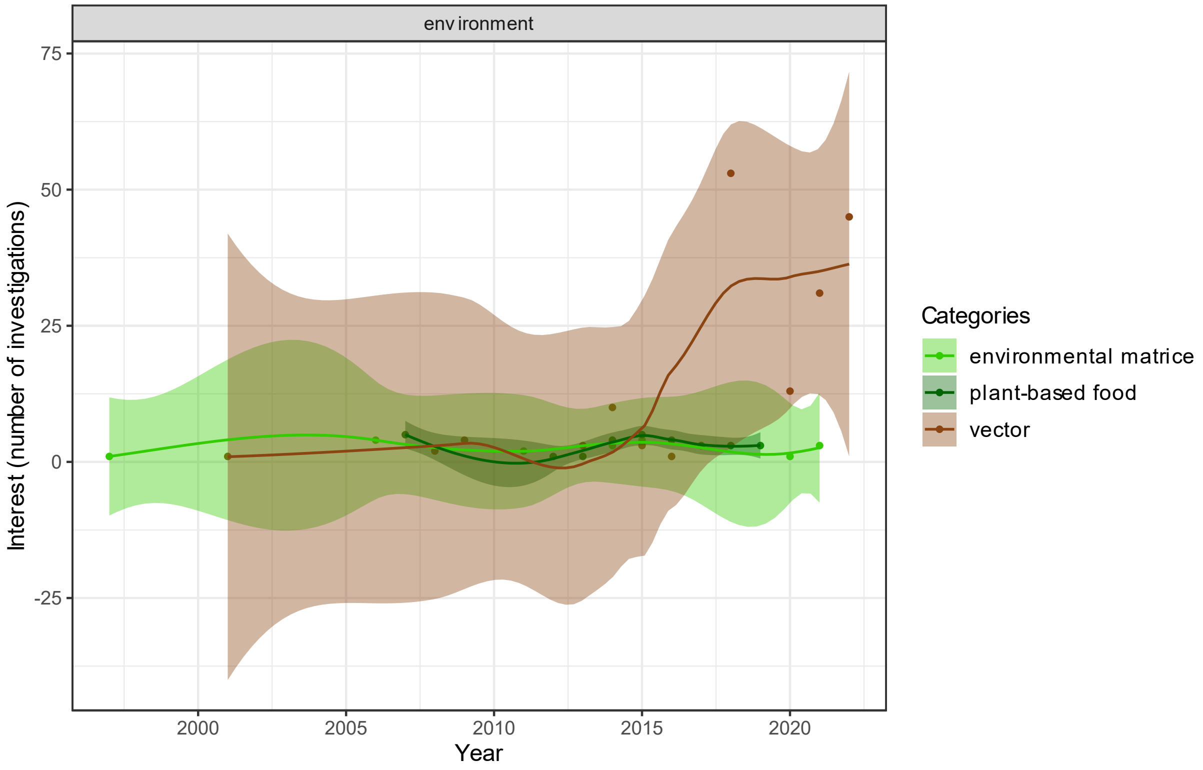

Supplementary Figure 5: Naturally occurring zoonotic agents that were directly or indirectly evidenced in Austria, 1975-2022. The colours correspond to the superkingdom (Bacteria = green, Viruses = pink, Eukaryota = orange, other = blue). Node size is proportional to the number of times a zoonotic agent was evidenced (and results published) between 1975 and 2022. All zoonotic agent names were resolved against the NCBI Taxonomy database, when this was not possible the most specific denomination of the zoonotic agent as mentioned in the publication was used.

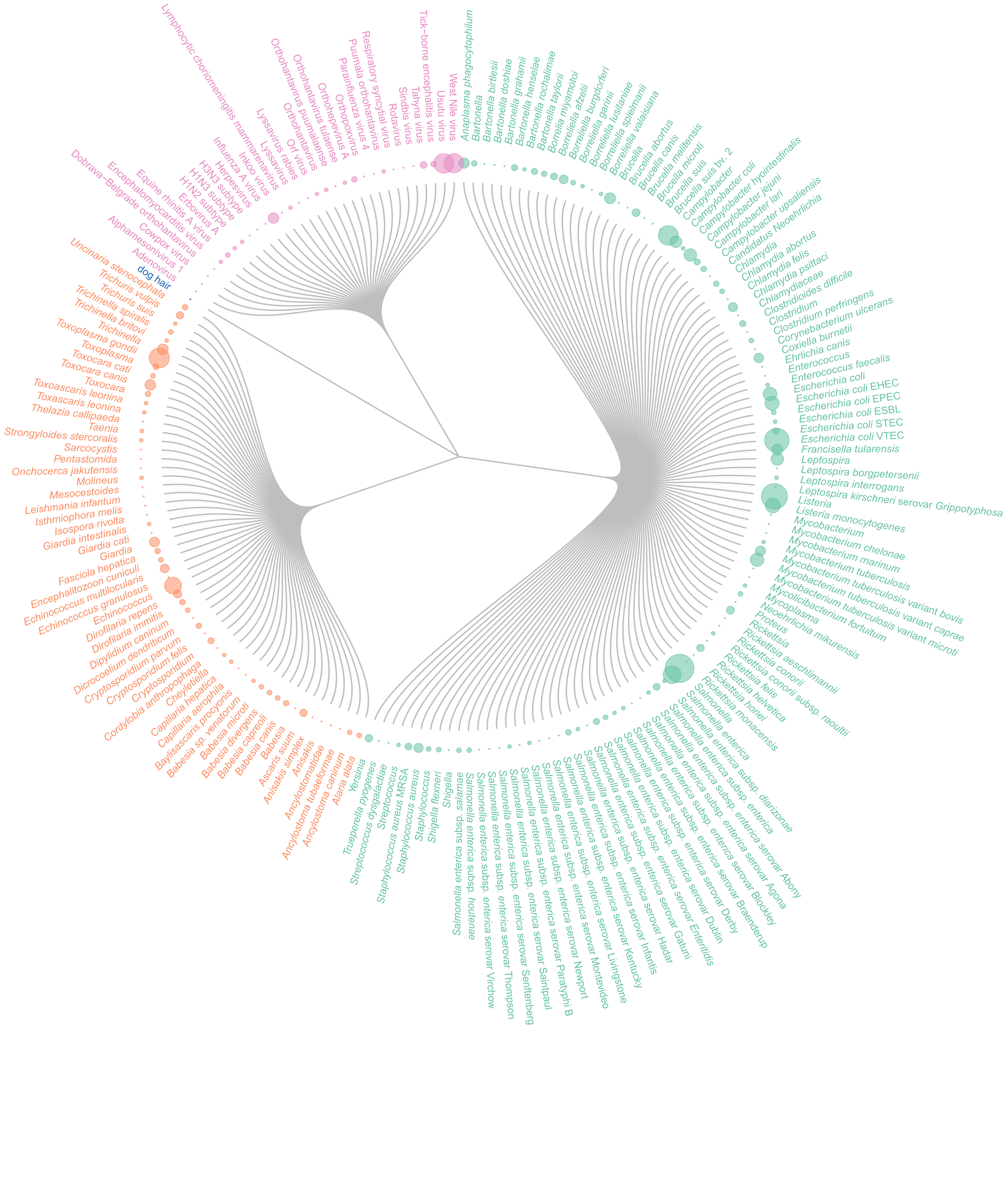

Supplementary Table 2: Taxonomic class, order, NCBI-resolved scientific and common names, and type of hosts, in which zoonotic agents were directly or indirectly evidenced in Austria between 1975 and 2022.

Host types are defined as follow: companion animal (defined as domesticated animals possessed by a person for reasons other than food or resource production, including domesticated small rodents or exotic companion animals), livestock (defined as domesticated animal kept for resource and food production), wildlife (defined as free-ranging or captive wild animal species that are not domesticated).

Host class	Host order	NCBI-resolved host scientific name[1]	NCBI-resolved host common name	Host type
Actinopteri	Salmoniformes	*Salmo trutta fario*	river trout	wildlife
Aves	Accipitriformes	*Accipiter gentilis*	Northern goshawk	wildlife
Aves	Accipitriformes	*Accipiter nisus*	Eurasian sparrowhawk	wildlife
Aves	Accipitriformes	*Accipitriformes*	hawks and eagles	wildlife
Aves	Accipitriformes	*Aegypius monachus*	Cinereous vulture	wildlife
Aves	Accipitriformes	*Aquila chrysaetos*	golden eagle	wildlife
Aves	Accipitriformes	*Aquila heliaca*	eastern imperial eagle	wildlife
Aves	Accipitriformes	*Aquila nipalensis*	steppe eagle	wildlife
Aves	Accipitriformes	*Buteo buteo*	common buzzard	wildlife
Aves	Accipitriformes	*Circus aeruginosus*	western marsh harrier	wildlife
Aves	Accipitriformes	*Gypaetus barbatus*	lammergeier	wildlife
Aves	Accipitriformes	*Haliaeetus albicilla*	white-tailed eagle	wildlife
Aves	Accipitriformes	*Neophron percnopterus*	Egyptian vulture	wildlife
Aves	Accipitriformes	*Pernis apivorus*	European honey-buzzard	wildlife
Aves	Anseriformes	*Anas*	ducks	wildlife
Aves	Anseriformes	*Anas platyrhynchos*	mallard	wildlife
Aves	Anseriformes	Anatidae	waterfowl	wildlife
Aves	Anseriformes	*Anser anser*	domestic goose	wildlife
Aves	Anseriformes	*Anser* sp.	goose	wildlife

Aves	Anseriformes	Anserinae	goose	wildlife
Aves	Anseriformes	*Cygnus*	swans	wildlife
Aves	Anseriformes	*Cygnus olor*	mute swan	wildlife
Aves	Apodiformes	*Apus apus*	common swift	wildlife
Aves	Charadriiformes	Charadriiformes	shorebirds and others	wildlife
Aves	Charadriiformes	Laridae	gulls	wildlife
Aves	Ciconiiformes	*Ciconia ciconia*	white stork	wildlife
Aves	Ciconiiformes	*Ciconia nigra*	black stork	wildlife
Aves	Ciconiiformes	Ciconiiformes	storks and others	wildlife
Aves	Columbiformes	Columbiformes	pigeons and others	wildlife
Aves	Columbiformes	*Streptopelia decaocto*	Eurasian collared-dove	wildlife
Aves	Coraciiformes	Coraciiformes	kingfisher	wildlife
Aves	Cuculiformes	Cuculiformes	cuckoos and others	wildlife
Aves	Falconiformes	*Falco*	falcons	wildlife
Aves	Falconiformes	*Falco rusticolus*	gyrfalcon	wildlife
Aves	Falconiformes	*Falco tinnunculus*	common kestrel	wildlife
Aves	Falconiformes	Falconiformes	falcons and others	wildlife
Aves	Galliformes	Galliformes	landfowls	wildlife
Aves	Galliformes	*Gallus gallus*	chicken	livestock
Aves	Galliformes	*Meleagris gallopavo*	turkey	livestock
Aves	Galliformes	*Pavo cristatus*	Indian peafowl	wildlife
Aves	Galliformes	Phasianinae	Phasianinae	wildlife
Aves	Galliformes	*Phasianus colchicus*	ring-necked pheasant	wildlife
Aves	Gruiformes	*Fulica*	coots	wildlife
Aves	Gruiformes	*Fulica atra*	Eurasian coot	wildlife

Aves	Gruiformes	Gruiformes	terrestrial and marshbirds	wildlife
Aves	Passeriformes	*Acrocephalus scirpaceus*	Eurasian reed warbler	wildlife
Aves	Passeriformes	*Coloeus monedula*	jackdaw	wildlife
Aves	Passeriformes	*Corvus cornix*	hooded crow	wildlife
Aves	Passeriformes	*Corvus corone*	carrion crow	wildlife
Aves	Passeriformes	*Corvus frugilegus*	rook	wildlife
Aves	Passeriformes	*Curruca communis*	greater whitethroat	wildlife
Aves	Passeriformes	*Curruca curruca*	lesser whitethroat	wildlife
Aves	Passeriformes	*Cyanistes caeruleus*	blue tit	wildlife
Aves	Passeriformes	*Delichon urbicum*	Northern house-martin	wildlife
Aves	Passeriformes	*Emberiza schoeniclus*	reed bunting	wildlife
Aves	Passeriformes	*Erithacus rubecula*	European robin	wildlife
Aves	Passeriformes	*Garrulus glandarius*	Eurasian jay	wildlife
Aves	Passeriformes	*Hirundo rustica*	barn swallow	wildlife
Aves	Passeriformes	*Panurus biarmicus*	bearded reedling	wildlife
Aves	Passeriformes	*Parus major*	great tit	wildlife
Aves	Passeriformes	*Passer domesticus*	house sparrow	wildlife
Aves	Passeriformes	*Passeriformes*	song birds	wildlife
Aves	Passeriformes	*Phoenicurus ochruros*	black redstart	wildlife
Aves	Passeriformes	*Pica pica*	Eurasian magpie	wildlife
Aves	Passeriformes	*Sitta europaea*	wood nuthatch	wildlife
Aves	Passeriformes	*Sylvia atricapilla*	blackcap	wildlife
Aves	Passeriformes	*Sylvia borin*	garden warbler	wildlife
Aves	Passeriformes	*Turdus merula*	blackbird	wildlife
Aves	Passeriformes	*Turdus philomelos*	song thrush	wildlife

Aves	Pelecaniformes	Ardeidae	herons	wildlife
Aves	Piciformes	*Dendrocopos major*	great spotted woodpecker	wildlife
Aves	Piciformes	Piciformes	woodpeckers and others	wildlife
Aves	Psittaciformes	*Nestor notabilis*	kea	wildlife
Aves	Strigiformes	*Asio otus*	long-eared owl	wildlife
Aves	Strigiformes	*Athene noctua*	little owl	wildlife
Aves	Strigiformes	*Bubo bubo*	Eurasian eagle-owl	wildlife
Aves	Strigiformes	*Bubo scandiacus*	snowy owl	wildlife
Aves	Strigiformes	*Strigiformes*	owls	wildlife
Aves	Strigiformes	*Strix aluco*	tawny owl	wildlife
Aves	Strigiformes	*Strix nebulosa*	Great grey owl	wildlife
Aves	Strigiformes	*Strix uralensis*	Ural owl	wildlife
Aves	Strigiformes	*Tyto alba*	barn owl	wildlife
Lepidosauria	Squamata	*Boa constrictor*	boa	companion animal
Lepidosauria	Squamata	*Brachylophus fasciatus*	banded iguana	companion animal
Lepidosauria	Squamata	*Chamaeleo calyptratus*	veiled chameleon	companion animal
Lepidosauria	Squamata	*Corallus hortulanus*	garden tree boa	companion animal
Lepidosauria	Squamata	*Cyclura cornuta*	rhinocerus iguana	companion animal
Lepidosauria	Squamata	*Cyclura cychlura*	NA	companion animal
Lepidosauria	Squamata	*Cyclura nubila*	NA	companion animal
Lepidosauria	Squamata	*Cyclura rileyi*	NA	companion animal
Lepidosauria	Squamata	*Epicrates cenchria*	NA	companion animal
Lepidosauria	Squamata	*Furcifer pardalis*	NA	companion animal
Lepidosauria	Squamata	*Heterodon nasicus*	NA	companion animal
Lepidosauria	Squamata	*Iguana iguana*	common green iguana	companion animal

Lepidosauria	Squamata	*Lampropeltis alterna*	NA	companion animal
Lepidosauria	Squamata	*Lampropeltis mexicana*	NA	companion animal
Lepidosauria	Squamata	*Lampropeltis ruthveni*	NA	companion animal
Lepidosauria	Squamata	*Lampropeltis triangulum campbelli*	NA	companion animal
Lepidosauria	Squamata	*Lampropeltis triangulum hondurensis*	NA	companion animal
Lepidosauria	Squamata	*Morelia spilota*	carpet python	companion animal
Lepidosauria	Squamata	*Morelia viridis*	green tree python	companion animal
Lepidosauria	Squamata	*Phelsuma madagascariensis*	Madagascar day gecko	companion animal
Lepidosauria	Squamata	*Physignathus cocincinus*	NA	companion animal
Lepidosauria	Squamata	*Pogona vitticeps*	central bearded dragon	companion animal
Lepidosauria	Squamata	*Python molurus*	Indian rock python	companion animal
Lepidosauria	Squamata	*Python regius*	NA	companion animal
Lepidosauria	Squamata	*Sanzinia madagascariensis*	NA	companion animal
Lepidosauria	Squamata	*Sceloporus malachiticus*	NA	companion animal
Lepidosauria	Squamata	Serpentes	snakes	companion animal
Lepidosauria	Squamata	*Tropidophorus grayi*	NA	companion animal
Lepidosauria	NA	lizard (unspecified)	lizard (unspecified)	companion animal
Mammalia	Artiodactyla	*Bos taurus*	cattle	livestock
Mammalia	Artiodactyla	*Capra hircus*	goat	livestock
Mammalia	Artiodactyla	*Capra ibex*	Alpine ibex	wildlife
Mammalia	Artiodactyla	*Capreolus capreolus*	Western roe deer	wildlife
Mammalia	Artiodactyla	Cervidae	deer	wildlife
Mammalia	Artiodactyla	*Cervus elaphus*	red deer	wildlife
Mammalia	Artiodactyla	*Dama dama*	fallow deer	wildlife
Mammalia	Artiodactyla	*Lama glama*	llama	livestock

Mammalia	Artiodactyla	*Ovis aries*	sheep	livestock
Mammalia	Artiodactyla	*Ovis aries musimon*	mouflon	wildlife
Mammalia	Artiodactyla	*Rupicapra rupicapra*	chamois	wildlife
Mammalia	Artiodactyla	*Sus scrofa*	pig	livestock
Mammalia	Artiodactyla	*Sus scrofa*	pig	wildlife
Mammalia	Artiodactyla	*Sus scrofa* (w)	wild boar	wildlife
Mammalia	Carnivora	*Canis lupus familiaris*	dog	companion animal
Mammalia	Carnivora	*Felis catus*	domestic cat	companion animal
Mammalia	Carnivora	*Nyctereutes procyonoides*	raccoon dog	wildlife
Mammalia	Carnivora	*Procyon lotor*	raccoon	wildlife
Mammalia	Carnivora	*Vulpes*	Vulpes	wildlife
Mammalia	Carnivora	*Vulpes vulpes*	red fox	wildlife
Mammalia	Eulipotyphla	*Erinaceus europaeus*	western European hedgehog	wildlife
Mammalia	Eulipotyphla	*Sorex araneus*	European shrew	wildlife
Mammalia	Lagomorpha	*Lepus*	hares	wildlife
Mammalia	Lagomorpha	*Lepus europaeus*	European hare	wildlife
Mammalia	Lagomorpha	*Lepus timidus*	mountain hare	wildlife
Mammalia	Lagomorpha	*Oryctolagus cuniculus*	rabbit	companion animal
Mammalia	Perissodactyla	*Equus caballus*	horse	companion animal
Mammalia	Primates	*Homo sapiens*	human	human
Mammalia	Primates	*Macaca fuscata*	Japanese macaque	wildlife
Mammalia	Primates	*Macaca sylvanus*	Barbary ape	wildlife
Mammalia	Primates	*Pongo pygmaeus*	Bornean orangutan	wildlife
Mammalia	Rodentia	*Apodemus agrarius*	Eurasian field mouse	wildlife
Mammalia	Rodentia	*Apodemus flavicollis*	Yellow-necked field mouse	wildlife

Mammalia	Rodentia	*Apodemus sylvaticus*	European woodmouse	wildlife
Mammalia	Rodentia	*Arvicola amphibius*	Eurasian water vole	wildlife
Mammalia	Rodentia	*Castor fiber*	Eurasian beaver	wildlife
Mammalia	Rodentia	*Marmota marmota*	European marmot	wildlife
Mammalia	Rodentia	*Microtus arvalis*	common vole	wildlife
Mammalia	Rodentia	*Mus musculus*	house mouse	wildlife
Mammalia	Rodentia	*Myodes glareolus*	bank vole	wildlife
Mammalia	Rodentia	*Rattus norvegicus*	Norway rat	wildlife
Mammalia	NA	new-world camelid (unspecified)	new-world camelid (unspecified)	livestock
Testudinata	Testudines	Testudines	turtles	companion animal
Testudinata	Testudines	Testudinidae	tortoises	companion animal
Testudinata	Testudines	*Testudo hermanni*	NA	companion animal

[1] The most specific denomination retrieved from the publication is used when NCBI validation was not possible.

Supplementary Table 3: Zoonotic agents evidenced in invertebrate vectors, Austria, 1975-2022.

NCBI-resolved vector name	Vector taxonomic order	Vector taxonomic family	NCBI-resolved zoonotic agent name
Aedes vexans	Diptera	Culicidae	Tahyna virus
Culex[1]	Diptera	Culicidae	Tahyna virus<br>West Nile virus
Culex pipiens	Diptera	Culicidae	West Nile virus
Uranotaenia unguiculata	Diptera	Culicidae	Alphamesonivirus 1
Dermacentor reticulatus	Ixodida	Ixodidae	*Bartonella*[1]<br>*Francisella tularensis*<br>*Rickettsia conorii* subsp. *raoultii*<br>*Rickettsia helvetica*
Haemaphysalis concinna	Ixodida	Ixodidae	*Bartonella*[1]
Haemaphysalis inermis	Ixodida	Ixodidae	*Bartonella*[1]<br>*Rickettsia helvetica*
Hyalomma[1]	Ixodida	Ixodidae	*Rickettsia*[1]
Hyalomma marginatum	Ixodida	Ixodidae	*Rickettsia aeschlimannii*
Ixodes[1]	Ixodida	Ixodidae	*Rickettsia helvetica*
Ixodes ricinus	Ixodida	Ixodidae	*Anaplasma phagocytophilum*<br>*Babesia divergens*<br>*Babesia microti*<br>*Babesia sp. venatorum*<br>*Bartonella*[1]<br>*Borrelia miyamotoi*<br>*Borreliella afzelii*<br>*Borreliella burgdorferi*<br>*Borreliella garinii*<br>*Borreliella lusitaniae*<br>*Borreliella spielmanii*<br>*Borreliella valaisiana*<br>*Neoehrlichia mikurensis*<br>*Rickettsia*[1]<br>*Rickettsia helvetica*<br>*Rickettsia monacensis*
Ctenocephalides felis	Siphonaptera	Pulicidae	*Bartonella henselae*<br>*Rickettsia felis*

[1] Most specific denomination retrieved from the publication.

Supplementary Table 4: Zoonotic agents evidenced in environmental matrices, Austria, 1975-2022.

Environmental matrix	NCBI-resolved zoonotic agent name
Food processing plant	*Listeria monocytogenes*
Meat processing plant	*Salmonella*[1]<br>*Salmonella enterica* subsp. *enterica* serovar *Braenderup*<br>*Salmonella enterica* subsp. *enterica serovar Infantis*<br>*Salmonella enterica* subsp. *enterica* serovar Livingstone
Public lavatory	*Listeria monocytogenes*
Sandbox	*Toxocara*[1]
Slaughter knife	*Escherichia coli* EHEC[2]<br>*Escherichia coli* EPEC[3]
Water	*Cryptosporidium*[1]<br>*Giardia*[1]<br>*Mycobacterium*[1]

[1] Most specific denomination retrieved from the publication.

[2] Enterohaemorrhagic *E. coli.*

[3] Enteropathogenic *E. coli.*

Supplementary Figure 6: Alluvial plot of the zoonotic agents found in food products, Austria, 1975-2022.

The zoonotic agent´s NCBI-resolved genus is shown on the first axis, the food origin is displayed on the middle axis, and the food category is specified on the right axis. The y-axis represents the number of times an investigation yielded a positive result in the selected publications. The colours highlight the contamination of specific foodstuffs by three zoonotic agent genera, with red: *Salmonella*, purple: *Echinococcus*, and orange: *Trichinella.*

A total of 30 publications investigated food products. These products originated from 23 different food sources, encompassing plant-based food (n=9 publications, 5.6% of the studies investigating food products) and food from animal origin (n=30 studies), such as livestock- and horse-derived food, game-derived food products, and marine products (e.g., herring, mackerel, salmon, trout). Only one publication investigated honey, imported to Austria from Turkey and Egypt. The honey was investigated for *Listeria monocytogenes*, *Salmonella*, *Campylobacter,* and *Escherichia coli*. Results were negative.

The type of food that yielded most positive results were "meat and meat products" (55.8%). In 44.2% of the investigations, the animal origin of the investigated food was unspecified.

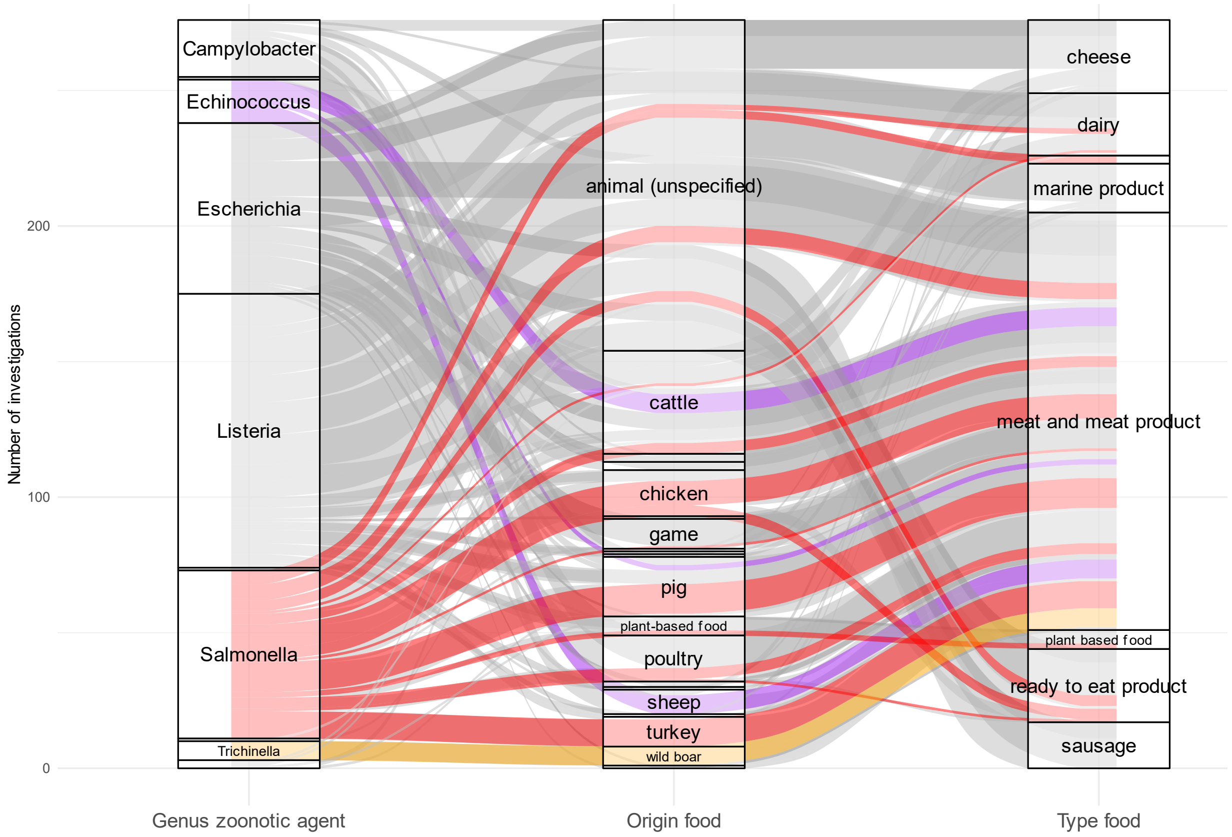

Supplementary Figure 7: Conventional representation of the bipartite network of zoonotic interactions (“zoonotic web”), Austria, 1975-2022.

The lower set of nodes represents zoonotic agents, while the upper set represents zoonotic sources categorised into hosts (red), vectors (yellow), foodstuffs (blue), and environmental matrices (green). The top 10 sources and zoonotic agents, based on node degree centrality, are highlighted with colours corresponding to their node category. Edges between the top 10 sources (coloured) and the zoonotic agents they interact with (in bold) are highlighted using the colour of the respective zoonotic source.

We opted for the D3 forceLink layout in the main article. This choice is based on its ability to effectively showcase zoonotic sources and agents, spatially grouping them and offering epidemiological insights into zoonotic dynamics. However, when it comes to representing the zoonotic web, a more conventional bipartite network can also be employed, similar to how a food web is typically depicted.

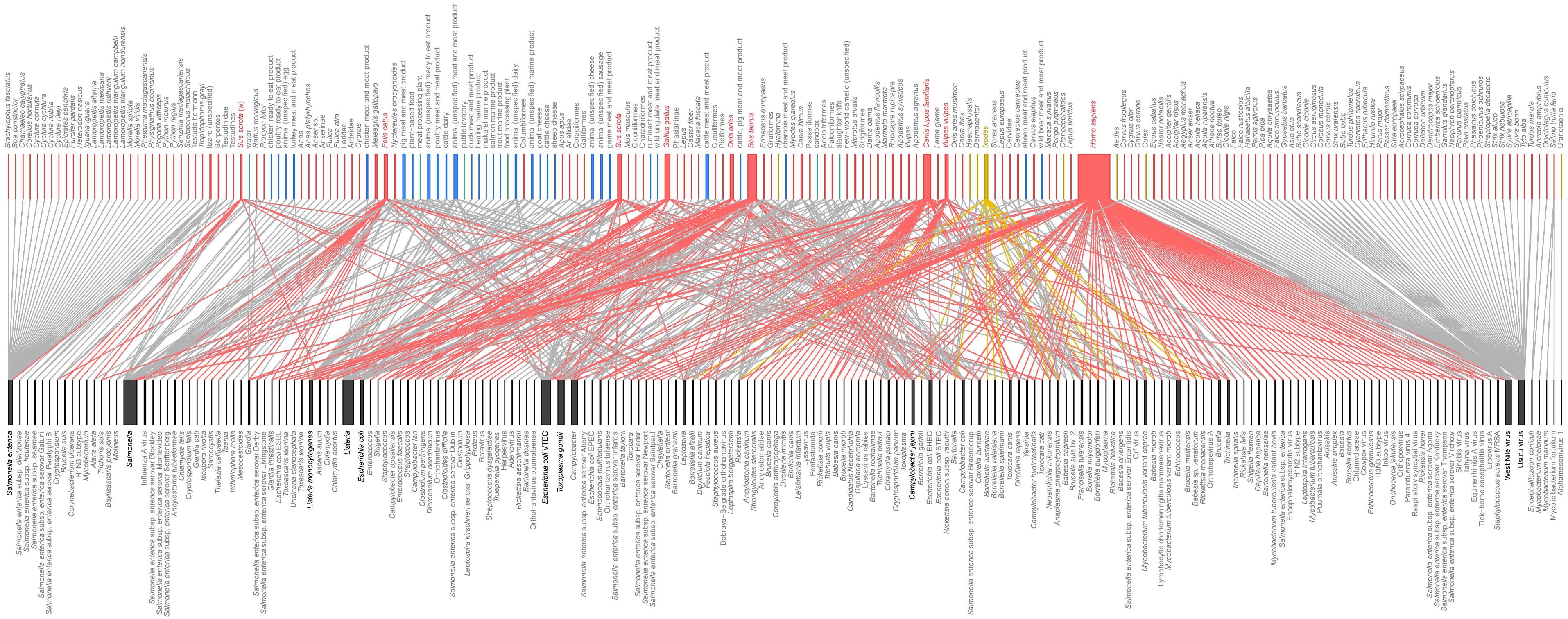

Supplementary Table 5: Kendall's Tau correlation coefficient of the four node centrality metrics in the scientific research effort-adjusted network of zoonotic agent sharing. Statistics shown is a two-sided Kendall rank correlation coefficient with adjustments for rank ties. All p-values are < 0.001. The normalised values of the weighted betweenness and closeness were used.

	**Degree centrality**	**Strength**	**Betweenness centrality**	**Closeness centrality**
**Degree centrality**	1	0.774	0.371	0.260
**Strength**		1	0.313	0.262
**Betweenness centrality**			1	0.414
**Closeness centrality**				1

Supplementary Table 6: Pairwise comparisons of the node centrality metrics per category of zoonotic sources using a two-sided Wilcoxon rank sum test. The table reports the p-values that were adjusted following the Benjamini-Hochberg procedure. A p-value ≤ 0.05 was considered significant, indicating a difference between average values of the centrality metrics between two groups of nodes. The Kruskall-Wallis test revealed no difference in the betweenness centrality between the four zoonotic sources.

**Degree centrality**			
	Vectors	Food matrices	Environmental matrices
Hosts	0.013	0.150	0.013
Vectors		0.025	0.667
Food matrices			0.031
**Strength**			
Hosts	0.003	0.562	0.017
Vectors		0.005	0.354
Food matrices			0.017
**Closeness centrality**			
Hosts	0.078	9.3e-05	0.816
Vectors		0.009	0.217
Food matrices			0.025

Supplementary Table 7: Composition and size of the zoonotic agent sharing communities as detected by the Leiden algorithm. The community number corresponds to the colour in Figure 4b.

Community number (size)	Community members (alphabetical order)
1 (n=51)	*Aedes ; Apodemus agrarius ; Apodemus flavicollis ; Apodemus sylvaticus ; Bos taurus ; Canis lupus familiaris ; Capra hircus ; Capra ibex ; Capreolus capreolus ; Castor fiber ;* Cervidae *; Cervus elaphus ; Corvus frugilegus ; Ctenocephalides ; Cygnus olor ; Dama dama ; Dermacentor ; Erinaceus europaeus ; Felis catus ; Haemaphysalis ; Homo sapiens ; Hyalomma ; Ixodes ; Lama glama ; Lepus ; Lepus europaeus ; Lepus timidus ; Macaca fuscata ; Macaca sylvanus ; Marmota marmota ; Microtus arvalis ; Mus musculus ; Myodes glareolus ;* new-world camelid (unspecified) ; *Nyctereutes procyonoides ; Ovis aries ; Ovis aries musimon ;* Phasianinae ; *Pongo pygmaeus ; Procyon lotor ; Rattus norvegicus ; Rupicapra rupicapra ;* sandbox ; sheep meat and meat product ; slaughter knife *; Sorex araneus ; Sus scrofa ; Vulpes ; Vulpes vulpes ;* water ; wild boar meat and meat product.
2 (n=33)	*Boa constrictor ; Brachylophus fasciatus ; Chamaeleo calyptratus ; Corallus hortulanus ; Cyclura cornuta ; Cyclura cychlura ; Cyclura nubila ; Cyclura rileyi ; Epicrates cenchria ; Furcifer pardalis ; Heterodon nasicus ; Iguana iguana , Lampropeltis alterna ; Lampropeltis mexicana , Lampropeltis ruthveni ; Lampropeltis triangulum campbelli , Lampropeltis triangulum hondurensis ,* lizard (unspecified) ; *Morelia spilota ; Morelia viridis ; Phelsuma madagascariensis , Physignathus cocincinus , Pogona vitticeps , Python molurus , Python regius , Sanzinia madagascariensis ; Sceloporus malachiticus ,* Serpentes , *Sus scrofa* (wild boar) ; Testudines ; Testudinidae *; Testudo hermanni ; Tropidophorus grayi.*
3 (n=24)	Accipitriformes ; *Anas ; Anas platyrhynchos* ; Anatidae *; Anser* sp. ; Anserinae ; *Apus apus* ; Ardeidae ; Charadriiformes ; Ciconiiformes ; Columbiformes ; Coraciiformes ; Cuculiformes ; *Cygnus* ; Falconiformes ; *Fulica* ; *Fulica atra* ; Galliformes ; *Gallus gallus* ; Gruiformes ; Laridae ; Passeriformes ; Piciformes ; Strigiformes.
4 (n=33)	animal (unspecified) cheese ; animal (unspecified) dairy ; animal (unspecified) egg ; animal (unspecified) marine product ; animal (unspecified) meat and meat product ; animal (unspecified) ready to eat product ; animal (unspecified) sausage ; cattle cheese ; cattle dairy ; cattle meat and meat product ; cattle, pig meat and meat product ; chamois meat and meat product ; chicken meat and meat product ; chicken ready to eat product ; duck meat and meat product ; food processing plant ; game meat and meat product ; goat cheese ; herring marine product ; mackerel marine product ; meat processing plant ; *Meleagris gallopavo* ; pig meat and meat product ; plant-based food ; poultry meat and meat product ; poultry ready to eat product ; public lavatory ; ruminant meat and meat product ; salmon marine product ; sheep cheese ; trout marine product ; turkey meat and meat product ; wild ungulate meat and meat product.
5 (n=27)	*Accipiter gentilis ; Accipiter nisus ; Aegypius monachus ; Anser anser ; Aquila chrysaetos ; Aquila heliaca ; Aquila nipalensis ; Asio otus ; Athene noctua ; Bubo scandiacus ; Buteo buteo ; Ciconia ciconia ; Ciconia nigra ; Circus aeruginosus ; Coloeus monedula ; Corvus cornix ; Corvus corone ; Culex , Equus caballus ; Falco , Falco rusticolus , Gypaetus barbatus , Haliaeetus albicilla ; Nestor notabilis ; Pernis apivorus , Pica pica , Strix uralensis.*
6 (n=28)	*Acrocephalus scirpaceus ; Bubo bubo ; Curruca communis ; Curruca curruca ; Cyanistes caeruleus ; Delichon urbicum ; Dendrocopos major ; Emberiza schoeniclus ; Erithacus rubecula ; Falco tinnunculus ; Garrulus glandarius ; Hirundo rustica ; Neophron percnopterus , Panurus biarmicus ; Parus major ; Passer domesticus ; Pavo cristatus ; Phasianus colchicus ; Phoenicurus ochruros ; Sitta europaea ; Streptopelia decaocto ; Strix aluco ; Strix nebulosa ; Sylvia atricapilla ; Sylvia borin ; Turdus merula ; Turdus philomelos ; Tyto alba.*

Supplementary Figure 8: Heatmap showing the circulating zoonotic agents within each zoonotic source community. Communities were determined using the Leiden algorithm run on the zoonotic agent sharing network, which edge weights represented the number of zoonotic agents shared, adjusted for the scientific research effort.

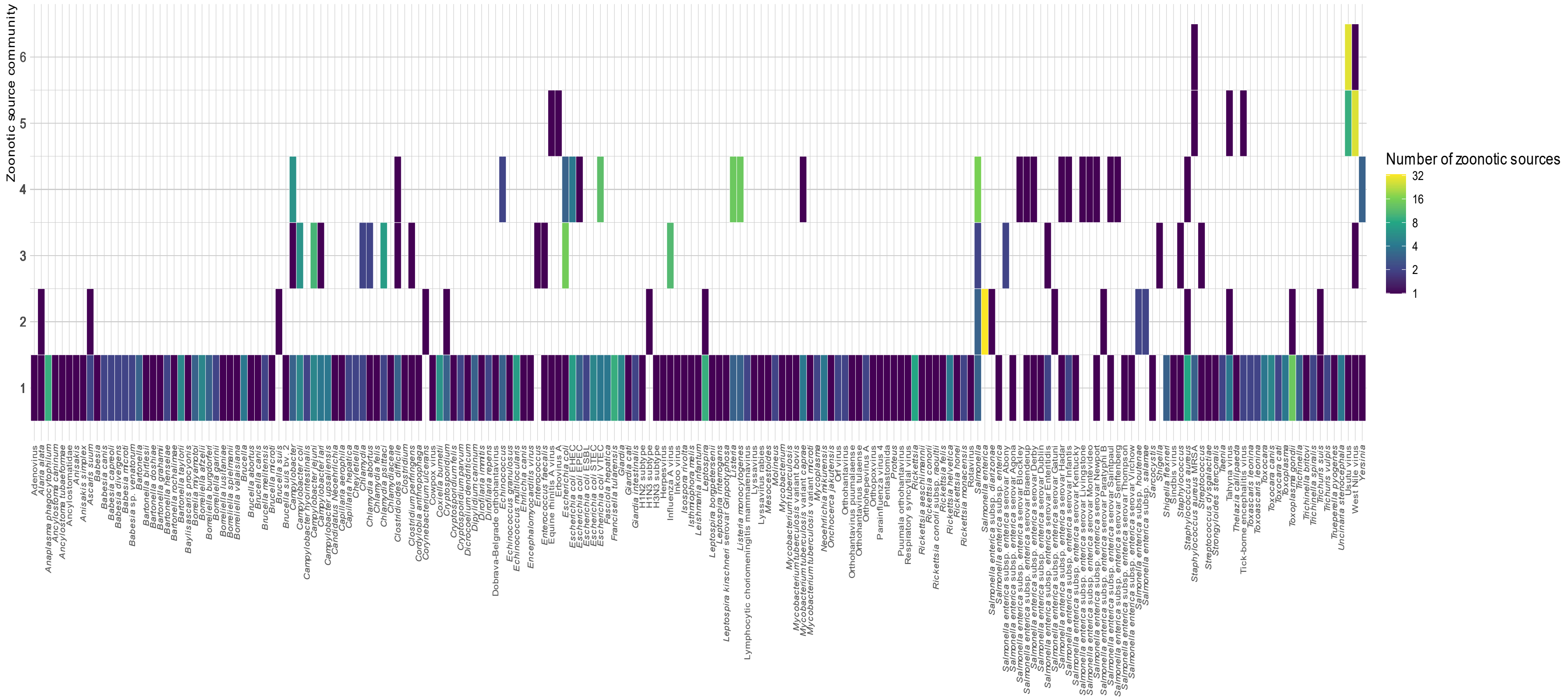

Supplementary Table 8: Importation events of zoonotic agents documented in Austria, 1975-2022. The publications underwent screening to identify any references to the potential acquisition of zoonotic agents from sources outside of Austria. Among the selected publications, eleven (4.5%) clearly affirmed that the zoonotic agents were not imported, whereas 14 (5.6 %) either expressed uncertainty about importation or acknowledged the possibility. Importation of zoonotic agents was confirmed in 21 (8.5%) publications, which results are presented in the table below. The table presents the unique combinations of zoonotic agents / probable origin(s) as outlined in the publication. It also indicates the number of times each unique combination was extracted from the selected publications (NA: information not available).

NCBI-resolved zoonotic agent name	Suspected country of origin	Number of times importation was evidenced
Anisakis simplex	Alaska	1
Babesia microti	USA	1
Brucella[1]	NA	8
Brucella melitensis	NA	2
Cordylobia anthropophaga	Gambia	1
Dirofilaria immitis	USA, Spain, Hungary, Greece	1
Dirofilaria repens	USA, Spain, Hungary, Greece	1
Echinococcus[1]	NA	7
Ehrlichia canis	Greece, Hungary, Spain, Italy, Portugal, France, USA, Asia, Argentina	1
Escherichia coli	Ethiopia, Nigeria, South Africa	1
Escherichia coli	Turkey, Egypt, China, United Arab Emirates, Russia, other	2
Escherichia coli VTEC[2]	Turkey, Egypt, China, United Arab Emirates, Russia, other	2
Leishmania infantum	Greece, Hungary, Spain, Italy, Portugal, Croatia, Africa, Asia	1
Leptospira[1]	South-East Asia, Africa, South America, Central Europe	1
Listeria monocytogenes	Mozambique, China, Turkey, United Arab Emirates, Albania,	1
Listeria monocytogenes	Turkey, Egypt, China, United Arab Emirates, Russia, other	2
Lyssavirus[1]	Serbia	1
Pentastomida[1]	USA	1
Rickettsia aeschlimannii	NA	1
Rickettsia conorii	Greece, Spain, Italy, Africa	1
Salmonella[1]	Ethiopia, Nigeria, South Africa	1
Salmonella[1]	Turkey, Egypt, China, United Arab Emirates, Russia, other	2
Trichinella[1]	Bosnia Herzegovina	1
Trichinella[1]	NA	5
Trichinella spiralis	Bosnia	1
Trichinella spiralis	Poland	1

[1] Most specific denomination retrieved from the publication.

[2] Verotoxigenic *E. coli.*

Supplementary Figure 9: Timeline of zoonotic agent emergence in Austria, 1975-2022, as reported from the selected publications.

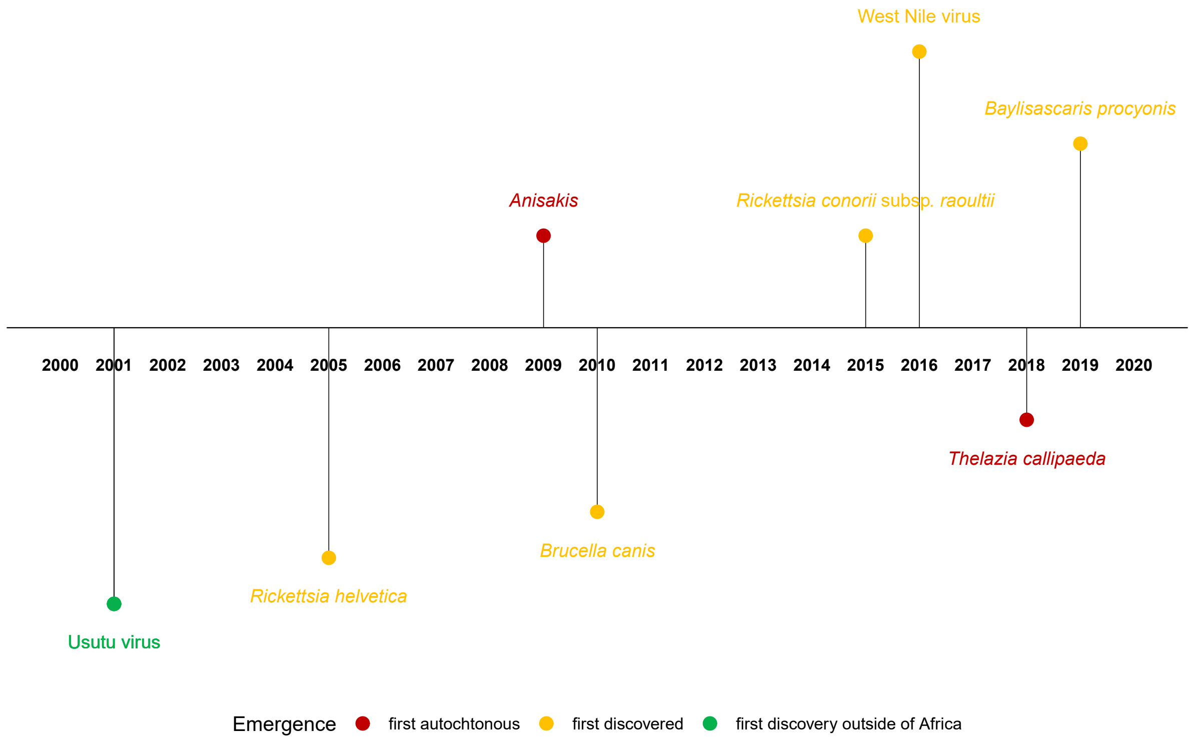

Supplementary Figure 10: Current source range of the eight zoonotic agents identified as emerging in Austria, 1975-2022. The subgraph was created by extracting from the zoonotic web the neighbourhood of the nodes representing emerging zoonotic agents (in bold) to the order 1. Node size represents the actor's degree in the original network and is coloured by the category the actor belongs to. Edge width (weight) represents the number of times the zoonotic host-agent association was reported in the selected publication (not adjusted). Zoonotic agents identified as emerging are shown in bold.

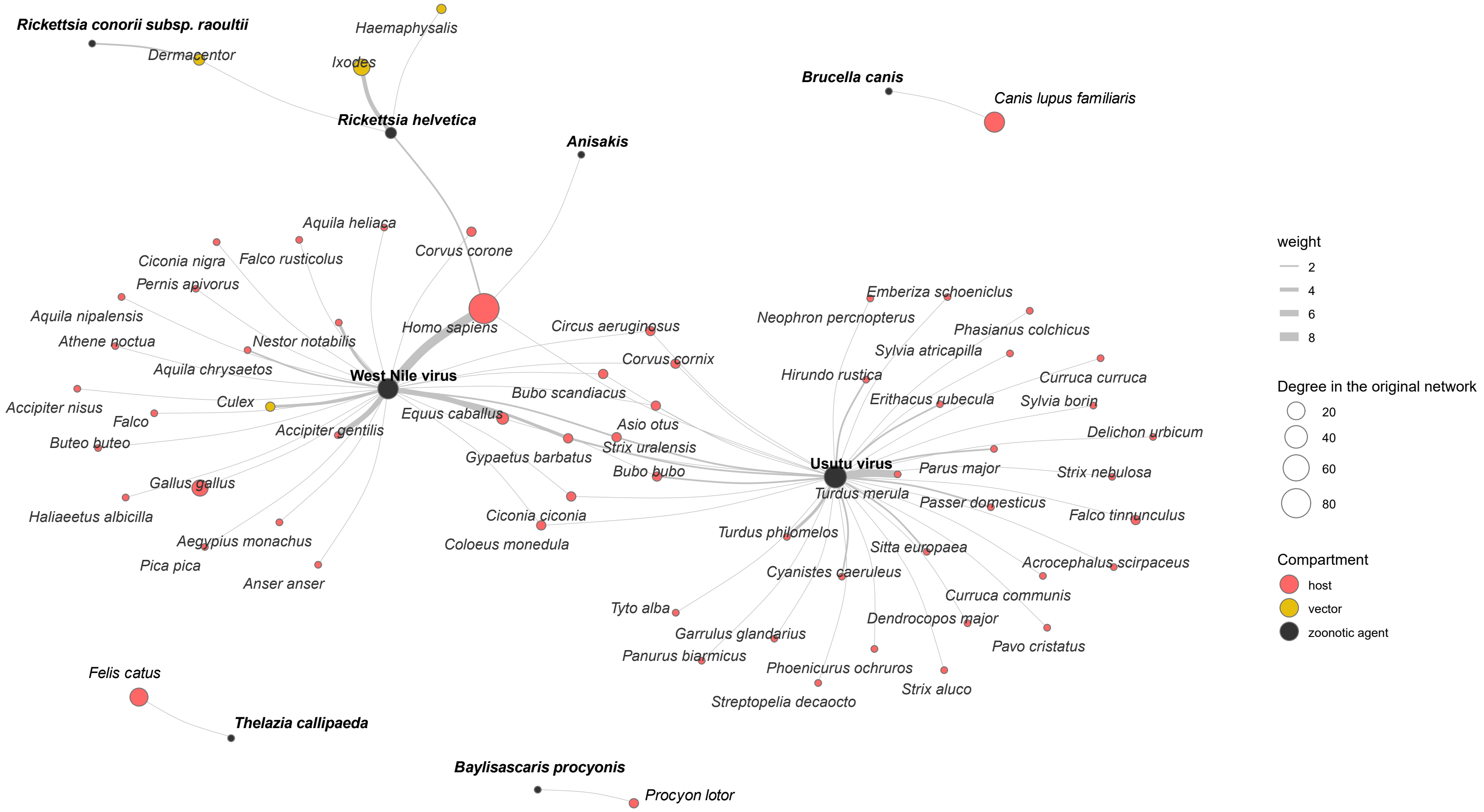

Supplementary Figure 11: Example of host subsystem (subgraph): subsystem "wild boar", illustrating a zoonotic source with high betweenness centrality. The subgraph was created by extracting from the zoonotic web the neighbourhood of the node *Sus scrofa* (w) (in bold), representing the wild boar, to the order 2. Node size represents the actor's degree in the original network and is coloured by the category the actor belongs to. Nodes with degree ≥ 5 are labelled. Edge width (weight) represents the number of times the zoonotic host-agent association was reported in the selected publications (not adjusted).

The wild boar (*Sus scrofa*) shares zoonotic agents with two host communities that have limited agent overlap, thereby acting as a bridge host (high betweenness centrality) for *Salmonella enterica* between 1) the community that encompasses human and most domesticated animals and 2) the community predominantly composed of reptiles and amphibians.

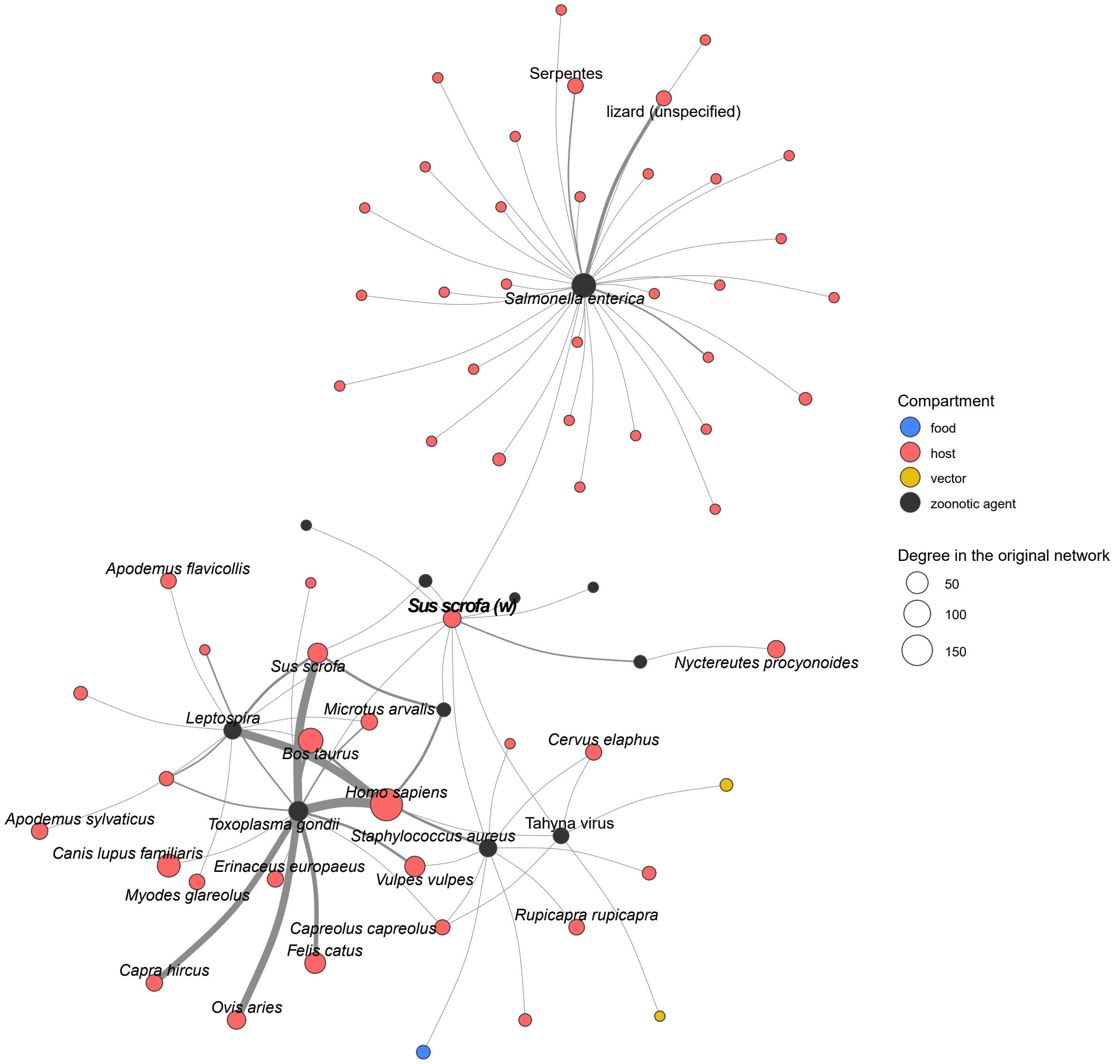

Supplementary Figure 12: Example of transmission chain: subsystem "*Listeria* sp.". The subgraph was created by extracting from the zoonotic web the neighbourhood of the nodes *Listeria* and *Listeria monocytogenes* (both in bold) to the order 1. Node size represents the actor's degree in the original network and is coloured by the category the actor belongs to. Edge width (weight) represents the number of times the zoonotic host-agent association was reported in the selected publications (not adjusted).

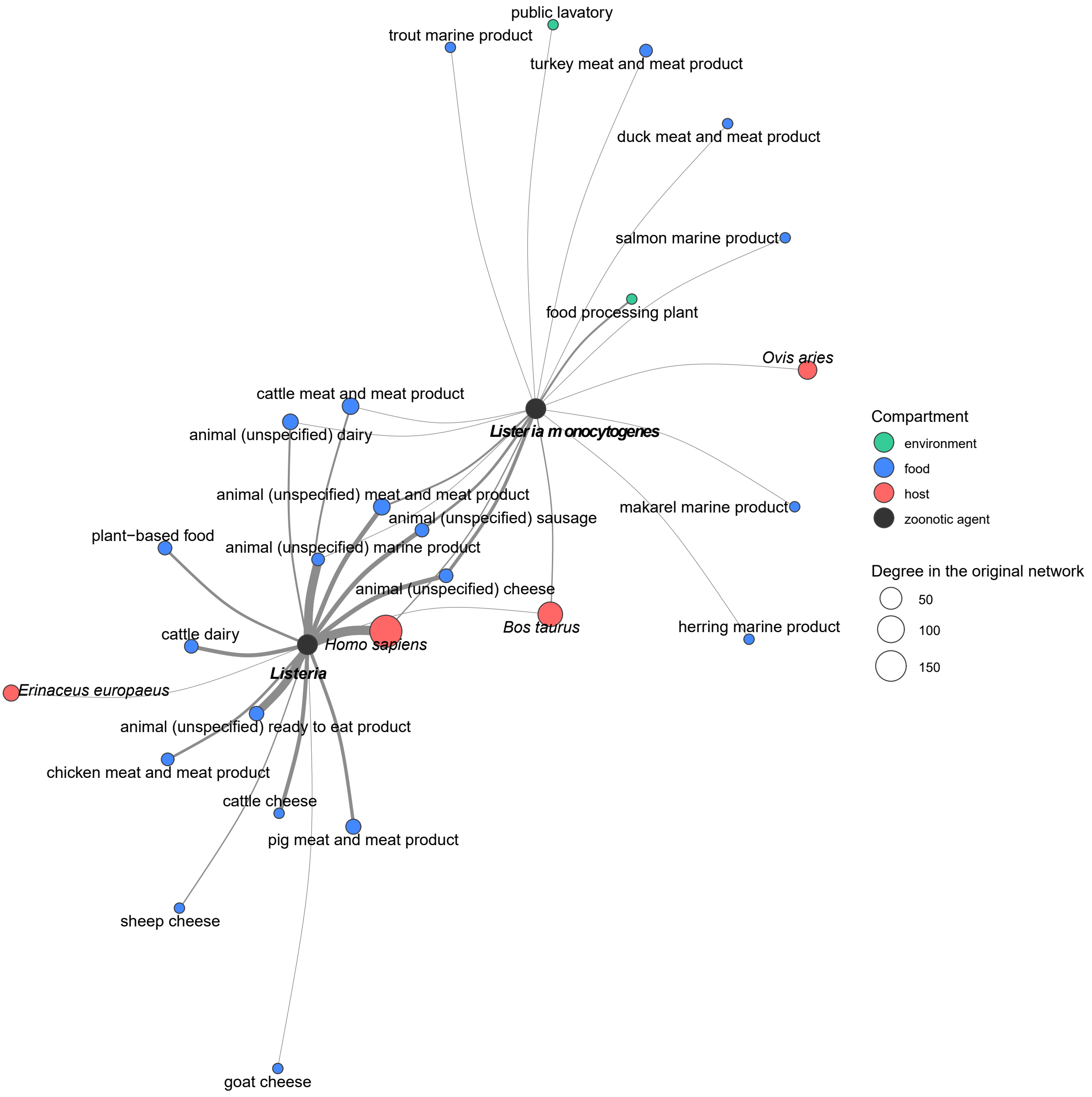

Supplementary Figure 13: Example of transmission chain: subsystem "shiga toxin-producing *E. coli*". The subgraph was created by extracting from the zoonotic web the neighbourhood of the nodes *Escherichia coli VTEC, Escherichia coli EHEC, Escherichia coli STEC* (in bold) to the order 1. Node size represents the actor's degree in the original network and is coloured by the category the actor belongs to. Edge width (weight) represent the number of times the zoonotic host-agent association was reported in the selected publications (not adjusted).

Shiga toxin-producing *E. coli* (STEC) strains could refer to both verotoxigenic *E. coli* (VTEC) and enterohaemorrhagic *E. coli* (EHEC) (Kaper, J. B., Nataro, J. P. & Mobley, H. L. Pathogenic *Escherichia coli*. *Nat. Rev. Microbiol*. 2, 123-140 (2004)). In our study, we have chosen a conservative approach to data validation/cleaning and preserved the authors´ terminology.

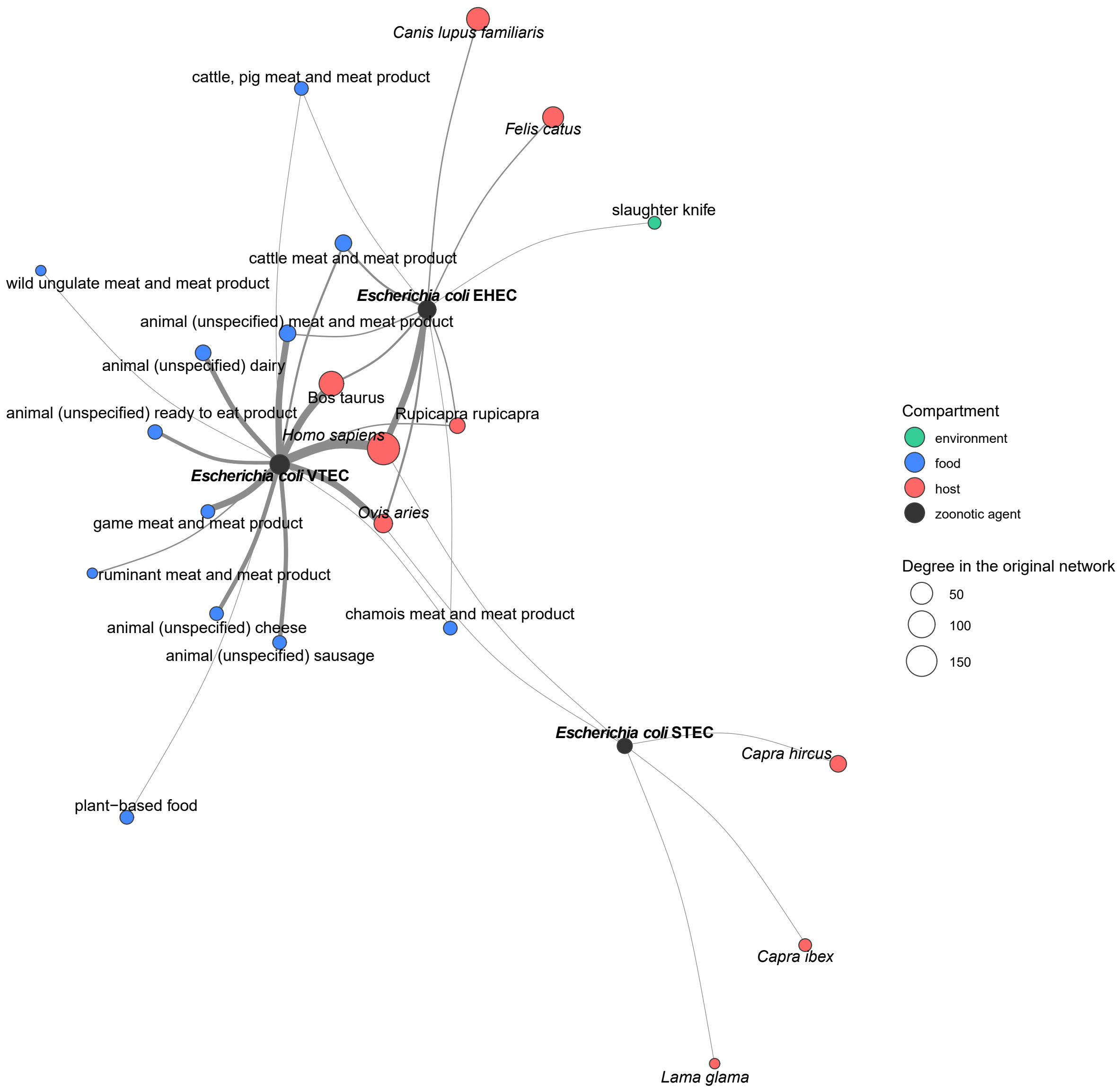

Supplement: Supplementary file 1 — Supplementary Information [file 41467_2024_49967_MOESM1_ESM.pdf]
