# Supplementary material for: A One Health framework for exploring zoonotic interactions demonstrated through a case study
Source: Nat Commun. 2024 Jul 15;15:5650. doi: 10.1038/s41467-024-49967-7 (PMC11250852; doi:10.1038/s41467-024-49967-7)
Supplement: Supplementary file 3 — Description of Additional Supplementary Files [file 41467_2024_49967_MOESM3_ESM.pdf]

Description of Additional Supplementary Files

File Name: Supplementary Code
Description: ZIP file containing the raw dataset generated in this study, its cleaned and validated version, the documented R scripts used for data cleaning, validation, processing, and analysis, and a READ.ME file, which contains the necessary instructions to run the code and replicate our results.
