# Supplementary material for: A One Health framework for exploring zoonotic interactions demonstrated through a case study
Source: Nat Commun. 2024 Jul 15;15:5650. doi: 10.1038/s41467-024-49967-7 (PMC11250852; doi:10.1038/s41467-024-49967-7)
Supplement: Supplementary file 5 — Reporting Summary [file 41467_2024_49967_MOESM5_ESM.pdf]

nature portfolio

Corresponding author(s): Amélie Desvars-Larrive

Last updated by author(s): Jun 17, 2024

Reporting Summary

Nature Portfolio wishes to improve the reproducibility of the work that we publish. This form provides structure for consistency and transparency in reporting. For further information on Nature Portfolio policies, see our Editorial Policies and the Editorial Policy Checklist.

Statistics

For all statistical analyses, confirm that the following items are present in the figure legend, table legend, main text, or Methods section.

n/a	Confirmed	
☐	☒	The exact sample size (*n*) for each experimental group/condition, given as a discrete number and unit of measurement
☒	☐	A statement on whether measurements were taken from distinct samples or whether the same sample was measured repeatedly
☐	☒	The statistical test(s) used AND whether they are one- or two-sided<br>*Only common tests should be described solely by name; describe more complex techniques in the Methods section.*
☐	☒	A description of all covariates tested
☐	☒	A description of any assumptions or corrections, such as tests of normality and adjustment for multiple comparisons
☐	☒	A full description of the statistical parameters including central tendency (e.g. means) or other basic estimates (e.g. regression coefficient) AND variation (e.g. standard deviation) or associated estimates of uncertainty (e.g. confidence intervals)
☒	☐	For null hypothesis testing, the test statistic (e.g. *F*, *t*, *r*) with confidence intervals, effect sizes, degrees of freedom and *P* value noted<br>*Give P values as exact values whenever suitable.*
☒	☐	For Bayesian analysis, information on the choice of priors and Markov chain Monte Carlo settings
☒	☐	For hierarchical and complex designs, identification of the appropriate level for tests and full reporting of outcomes
☒	☐	Estimates of effect sizes (e.g. Cohen's *d*, Pearson's *r*), indicating how they were calculated

Our web collection on statistics for biologists contains articles on many of the points above.

Software and code

Policy information about availability of computer code

Data collection: *No software was used. Data collection was performed manually.*

Data analysis: *All analyses were performed using R Statistical Software version 4.3.0 (2023-04-21 ucrt) within the interface RStudio 2023.06.0+421 (Release 583b465ecc45e60ee9de085148cd2f9741cc5214, 2023-06-05 for windows). We used the following R packages: ggplot2, dplyr, patchwork, stringr, ggfittext, tidyr, caret, matrixStats, plyr, egg, RColorBrewer, igraph, ggrepel, plyr, ggraph, bipartite, tidyverse, rsetse, hrbrthemes, lubridate, forcats, CINNA, bc3net, purrr, scales, ggalluvial, cowplot, colorspace.*

For manuscripts utilizing custom algorithms or software that are central to the research but not yet described in published literature, software must be made available to editors and reviewers. We strongly encourage code deposition in a community repository (e.g. GitHub). See the Nature Portfolio guidelines for submitting code & software for further information.

Data

Policy information about availability of data

All manuscripts must include a data availability statement. This statement should provide the following information, where applicable:

- Accession codes, unique identifiers, or web links for publicly available datasets
- A description of any restrictions on data availability
- For clinical datasets or third party data, please ensure that the statement adheres to our policy

All data supporting the findings of this study are available within the paper and its Supplementary Code. Furthermore, the collected data and the code that support

the findings of this study are available in Figshare with the identifier: https://figshare.com/s/c132cdb6a0575856ee90 (DOI: 10.6084/m9.figshare.25306177)

Research involving human participants, their data, or biological material

Policy information about studies with human participants or human data. See also policy information about sex, gender (identity/presentation), and sexual orientation and race, ethnicity and racism.

Reporting on sex and gender	NA
Reporting on race, ethnicity, or other socially relevant groupings	NA
Population characteristics	NA
Recruitment	NA
Ethics oversight	NA

Note that full information on the approval of the study protocol must also be provided in the manuscript.

Field-specific reporting

Please select the one below that is the best fit for your research. If you are not sure, read the appropriate sections before making your selection.

☐ Life sciences ☐ Behavioural & social sciences ☒ Ecological, evolutionary & environmental sciences

For a reference copy of the document with all sections, see nature.com/documents/nr-reporting-summary-flat.pdf

Life sciences study design

All studies must disclose on these points even when the disclosure is negative.

Sample size	NA
Data exclusions	NA
Replication	NA
Randomization	NA
Blinding	NA

Behavioural & social sciences study design

All studies must disclose on these points even when the disclosure is negative.

Study description	NA
Research sample	NA
Sampling strategy	NA
Data collection	NA
Timing	NA
Data exclusions	NA
Non-participation	NA
Randomization	NA

Ecological, evolutionary & environmental sciences study design

All studies must disclose on these points even when the disclosure is negative.

Study description	*Information about zoonotic agents and the sources investigated (found positive and negative) in Austria was searched through a systematic literature review and organized manually into a *.csv file. The data underwent cleaning and validation procedures in R. Notably, for each animal host, vector, and zoonotic agent, common and scientific names as well as taxonomic classification were resolved against the NCBI Taxonomy database using the R package taxize. The dataset was used to create an undirected network representing the web of naturally occurring zoonotic interactions, depicting the relationships between zoonotic actors. The zoonotic web was explored through network analysis. The zoonotic source-agent network was subsequently projected into a one-mode network of zoonotic agent sharing among sources. Edges were weighted by the number of shared zoonotic agents between two sources. To account for research biases, we considered, for each source, the total number of zoonotic investigations, following the method described by Gómez et al. (2013) and Luis et al. (2015). Node rankings through node centrality metrics were compared using the Kendall correlation test. Average values of the node centrality metrics were also compared between the four zoonotic source categories using the Kruskal-Wallis test. When a difference was evidenced, pairwise comparisons between zoonotic source categories were performed using the Wilcoxon rank sum test; p-values were adjusted following the Benjamini-Hochberg method. Moreover, we used the Leiden algorithm to detect communities of zoonotic agent sharing within the research-adjusted one-mode network of zoonotic sources. We subsequently characterized the circulating zoonotic agents within each source community. Finally, we investigated the circulation of zoonotic agents at human-animal-environment interfaces within the research effort-adjusted network of zoonotic agent sharing by searching and ranking the "One Health" 3-cliques in the network. Network analyses were performed using the R packages igraph and bipartite.*
Research sample	*The search identified 2,186 publications. After 542 duplicates were removed, 1,644 publications were screened with 1,269 excluded at the title/abstract screening stage as they were not eligible. This left 375 publications, of which 16 could not be retrieved, so that 359 full-text articles were assessed for eligibility, of which 229 met criteria for final inclusion. In addition, 17 publications were found in excluded review articles, leading to a total of 246 publications that were ultimately included in this study (168 scientific articles, 13 reports, and 65 theses).*<br>*The final dataset is a *.csv. file with 2,128 rows and 48 data fields. Each row represents one investigated zoonotic agent along with the results of the investigation in the animal host(s), vector(s), environmental or food matrix(-ices). The final dataset of zoonotic agents and sources investigated in Austria between 1975 and 2022 encompasses 227 zoonotic agents, 222 vertebrate hosts, 21 invertebrate vectors, 48 types of food samples, and 11 types of environmental samples.*<br>*Network analysis was performed on naturally occurring zoonotic interactions (i.e. the zoonotic agent was directly or indirectly evidenced in the zoonotic source), including 197 zoonotic agents, 155 distinct vertebrate hosts (including human, 111 wildlife, eight livestock, and 36 companion animal species), 12 different invertebrate (vector) species, 6 types of environmental media, and 31 categories of food.*
Sampling strategy	*The study does not involve a sampling procedure. All relevant information available in the literature was systematically collected and analyzed.*
Data collection	*Data collection was conducted manually by Anna Vogl.*<br><br>*Between 17 July and 23 August 2022, a systematic literature search was conducted according to the Preferred Reporting Items for Systematic Review and Meta-Analysis (PRISMA) guidelines, using the query ("Zoono*" AND ("Austria" OR "Österreich")) in the following databases: PubMed®, Scopus, and vetmed:seeker (internal database of the University of Veterinary Medicine Vienna, Austria), including articles published between the inception of the databases and the date of the search. Furthermore, the publication database of the AGES (https://www.ages.at/en/research/research-portal) was searched using the keyword "zoono". Titles and abstracts were first screened for relevance using the following inclusion criteria: the publication presented data pertaining to at least one zoonotic disease or agent that was investigated or documented in Austria and the agent was identified as zoonotic in the paper. Publications were excluded i) if they did not investigate or describe a zoonotic disease that was identified as such, ii) if research was not conducted in Austria, iii) if publications did not describe naturally occurring zoonotic infection, or iv) if publications described disease physiology or v) dealt with treatment or methods for pathogen detection. Book chapters, posters, literature reviews, statistical forecasts, and conference proceedings were excluded. Regarding antimicrobial resistant bacteria, papers were included if they specifically explored the animal-human interface and/or the authors referred to zoonotic transmission. To prevent duplication of data, diploma-, master's-, and doctorate thesis were not included if a peer-reviewed research paper published the same data.*<br>*In a second step, the full texts of the previously selected titles/abstracts were screened using the inclusion/exclusion criteria described above. Publications were excluded if they were not in German or English language or did not describe the situation in Austria. When a publication dealt with multiple countries, it was included if it provided specific information on zoonotic diseases in Austria.*<br>*The following data was extracted from the selected publications: i) Publication data: citation, year of publication, and type of publication; ii) Type of study: case study, original research, or national surveillance data; iii) Investigated zoonotic agent: agent type (e.g., bacterium, virus, parasite, fungus, prion, or other) and common/scientific names as mentioned in the information source; iv) Investigated host: host category, e.g., human, companion animal (defined as domesticated animals possessed by a person for reasons other than food or resource production, including domesticated small rodents or exotic companion animals), livestock (defined as domesticated animal kept for resource and food production), wildlife (defined as free-ranging or captive wild animal species that are not domesticated), common/scientific names as mentioned in the information source, if the zoonotic agent was detected in the host, i.e., seropositive (confirmed by the presence of antibodies), positive (direct detection of the agent), or negative; v) Investigated vector: common/scientific names as mentioned in the information source, and if the zoonotic agent was detected in the vector (positive/negative); vi) Investigated environmental matrix and if the zoonotic agent was detected in the matrix (positive/negative); vii) Investigated food matrix: the specific type of foodstuff investigated, the origin of the food product (animal or plant), and if the zoonotic agent was detected in the foodstuff (positive/negative); viii) Epidemiological context: study year, federal state(s), whether the case was imported and most probable origin, whether the zoonotic agent was mentioned as emerging in Austria, and whether specific*

	professional activities were deemed to carry an elevated risk of exposure.
Timing and spatial scale	*The dataset used in the study covers the period January 1975 (oldest publication retrieved from the systematic literature search) and 23 August 2022 (date of search). Geographic range of the dataset is Austria.*
Data exclusions	*After data was collected from the literature, no data was excluded from the analysis.*
Reproducibility	*The code underwent testing and execution by two different co-authors in R (ADL and GAP). Furthermore, a third author (LY) reproduced part of the findings in JavaScript language (Figure 3 and 5 for publication were produced in JavaScript). All findings (including figures) could be successfully replicated.*
Randomization	*The study does not include any experimental group and no randomization was necessary in our experimental design.*
Blinding	*The study does not include any allocation into groups as it is 100% computational. Therefore, blinding is not relevant.*

Did the study involve field work? ☐ Yes ☒ No

Field work, collection and transport

Field conditions	*NA*
Location	*NA*
Access & import/export	*NA*
Disturbance	*NA*

Reporting for specific materials, systems and methods

We require information from authors about some types of materials, experimental systems and methods used in many studies. Here, indicate whether each material, system or method listed is relevant to your study. If you are not sure if a list item applies to your research, read the appropriate section before selecting a response.

Materials & experimental systems

n/a	Involved in the study	
☒	☐	Antibodies
☒	☐	Eukaryotic cell lines
☒	☐	Palaeontology and archaeology
☒	☐	Animals and other organisms
☒	☐	Clinical data
☒	☐	Dual use research of concern
☒	☐	Plants

Methods

n/a	Involved in the study	
☒	☐	ChIP-seq
☒	☐	Flow cytometry
☒	☐	MRI-based neuroimaging

Antibodies

Antibodies used	*NA*
Validation	*NA*

Eukaryotic cell lines

Policy information about cell lines and Sex and Gender in Research

Cell line source(s)	*NA*
Authentication	*NA*
Mycoplasma contamination	*NA*
Commonly misidentified lines (See ICLAC register)	*NA*

Palaeontology and Archaeology

Specimen provenance	NA
Specimen deposition	NA
Dating methods	NA

☐ Tick this box to confirm that the raw and calibrated dates are available in the paper or in Supplementary Information.

Ethics oversight	NA

Note that full information on the approval of the study protocol must also be provided in the manuscript.

Animals and other research organisms

Policy information about studies involving animals; ARRIVE guidelines recommended for reporting animal research, and Sex and Gender in Research

Laboratory animals	NA
Wild animals	NA
Reporting on sex	NA
Field-collected samples	NA
Ethics oversight	NA

Note that full information on the approval of the study protocol must also be provided in the manuscript.

Clinical data

Policy information about clinical studies

All manuscripts should comply with the ICMJE guidelines for publication of clinical research and a completed CONSORT checklist must be included with all submissions.

Clinical trial registration	NA
Study protocol	NA
Data collection	NA
Outcomes	NA

Dual use research of concern

Policy information about dual use research of concern

Hazards

Could the accidental, deliberate or reckless misuse of agents or technologies generated in the work, or the application of information presented in the manuscript, pose a threat to:

No	Yes	
☒	☐	Public health
☒	☐	National security
☒	☐	Crops and/or livestock
☒	☐	Ecosystems
☒	☐	Any other significant area

Experiments of concern

Does the work involve any of these experiments of concern:

No	Yes	
☒	☐	Demonstrate how to render a vaccine ineffective
☒	☐	Confer resistance to therapeutically useful antibiotics or antiviral agents
☒	☐	Enhance the virulence of a pathogen or render a nonpathogen virulent
☒	☐	Increase transmissibility of a pathogen
☒	☐	Alter the host range of a pathogen
☒	☐	Enable evasion of diagnostic/detection modalities
☒	☐	Enable the weaponization of a biological agent or toxin
☒	☐	Any other potentially harmful combination of experiments and agents

Plants

Seed stocks	NA
Novel plant genotypes	NA
Authentication	NA

ChIP-seq

Data deposition

☐ Confirm that both raw and final processed data have been deposited in a public database such as GEO.

☐ Confirm that you have deposited or provided access to graph files (e.g. BED files) for the called peaks.

Data access links<br>*May remain private before publication.*	NA
Files in database submission	NA
Genome browser session<br>(e.g. UCSC)	NA

Methodology

Replicates	NA
Sequencing depth	NA
Antibodies	NA
Peak calling parameters	NA
Data quality	NA
Software	NA

Flow Cytometry

Plots

Confirm that:

- [ ] The axis labels state the marker and fluorochrome used (e.g. CD4-FITC).
- [ ] The axis scales are clearly visible. Include numbers along axes only for bottom left plot of group (a 'group' is an analysis of identical markers).
- [ ] All plots are contour plots with outliers or pseudocolor plots.
- [ ] A numerical value for number of cells or percentage (with statistics) is provided.

Methodology

Sample preparation	*NA*
Instrument	*NA*
Software	*NA*
Cell population abundance	*NA*
Gating strategy	*NA*

- [ ] Tick this box to confirm that a figure exemplifying the gating strategy is provided in the Supplementary Information.

Magnetic resonance imaging

Experimental design

Design type	*NA*
Design specifications	*NA*
Behavioral performance measures	*NA*

Acquisition

Imaging type(s)	*NA*
Field strength	*NA*
Sequence & imaging parameters	*NA*
Area of acquisition	*NA*

Diffusion MRI ☐ Used ☒ Not used

Preprocessing

Preprocessing software	*NA*
Normalization	*NA*
Normalization template	*NA*
Noise and artifact removal	*NA*
Volume censoring	*NA*

Statistical modeling & inference

Model type and settings	*NA*
Effect(s) tested	*NA*

Specify type of analysis: ☐ Whole brain ☐ ROI-based ☐ Both

Statistic type for inference

(See Eklund et al. 2016)

NA

Correction

NA

Models & analysis

n/a	Involved in the study
☐	☐ Functional and/or effective connectivity
☐	☐ Graph analysis
☐	☐ Multivariate modeling or predictive analysis

Functional and/or effective connectivity

NA

Graph analysis

NA

Multivariate modeling and predictive analysis

NA
